# A pre-ablative thyroid-stimulating hormone with 30–70 mIU/L achieves better response to initial radioiodine remnant ablation in differentiated thyroid carcinoma patients

**DOI:** 10.1038/s41598-020-80015-8

**Published:** 2021-01-14

**Authors:** Juan Xiao, Canhua Yun, Jingjia Cao, Shouluan Ding, Chunchun Shao, Lina Wang, Fengyan Huang, Hongying Jia

**Affiliations:** 1 Center of Evidence-Based Medicine, Institute of Medical Sciences, the Second Hospital, Cheeloo College of Medicine, Shandong University, Jinan, 250033 China; 2 Department of Nuclear Medicine, the Second Hospital, Cheeloo College of Medicine, Shandong University, Jinan, China; 3 Department of Laboratory Medicine, the Second Hospital, Cheeloo College of Medicine, Shandong University, Jinan, China; 4 School of Public Health, Cheeloo College of Medicine, Shandong University, Jinan, China

**Keywords:** Thyroid cancer, Thyroid cancer

## Abstract

Our aim was to clarify the optimum pre-ablative thyroid-stimulating hormone (TSH) level for initial radioiodine remnant ablation (RRA) in patients with differentiated thyroid carcinoma (DTC). From December 2015 to May 2019, 689 patients undergone RRA at Nuclear Medicine Department, Second Hospital of Shandong University were included in the study. Patients were categorized by their pre-ablative TSH level grouping of < 30, 30–70 and ≥ 70 mIU/L. Response to RRA were evaluated as complete response (including excellent and indeterminate response) and incomplete response (including biochemical and structural incomplete response) after a follow-up of 6–8 months. Multivariable binary logistic regression model was used to explore the optimum pre-ablative TSH level range and independent factors associated with response to RRA. Rates of complete response to RRA were 63.04%, 74.59% and 66.41% in TSH level groups of < 30, 30–70 and ≥ 70 mIU/L, separately. With multivariate analysis, the study found that pre-ablative TSH levels, gender and lymph node dissection were independent predictors of response to RRA. TSH between 30 and 70 mIU/L had a higher rate of complete response compared with TSH < 30 mIU/L, OR 0.451 (95% CI 0.215–0.958, *P* = 0.036). A pre-ablative TSH level of 30–70 mIU/L was appropriate for patients with DTC to achieve a better response to RRA.

## Introduction

The incidence of differentiated thyroid cancer (DTC) has risen rapidly worldwide in the last few decades^1^. Postoperative radioiodine remnant ablation (RRA) with ^131^I which can improve disease-free survival of DTC patients is widely used all over the world^2^. Thyrotropin stimulation before RRA has been a long-established standard of care. Since, it is well accepted that elevated serum thyroid-stimulating hormone (TSH) ensures maximize radioiodine uptake in thyroid remnants^3^. There are mainly two methods currently in use for ensuring elevated TSH level: (1) 3 to 4-week period of thyroxine withdrawal; (2) use of an intramuscular injection of recombinant TSH^4^. And in general, most medical institutions in China choose the treatment of thyroxine withdrawal.

A goal TSH of > 30 mIU/L has been generally adopted in preparation for RRA therapy, which was recommended by American Thyroid Association (ATA) guideline^5^. However, this recommendation is based on only one old observational research^3^, and there is uncertainty on the optimal level of pre-ablative TSH in considering outcome effects. Zhao et al. reported a pre-ablative TSH level of 90 to less than 120 mIU/L might achieve a better response to ^131^I therapy^6^. However, another study demonstrated that endogenous TSH levels at the time of ^131^I ablation were not related to the ablation success rates and long-term outcomes^7^. To clarify the optimal TSH level for initial RRA, we retrospectively evaluated the relationship between pre-ablative TSH level and response to ^131^I therapy of DTC patients.

## Methods

Patient data extracted from clinical electronic medical record system was deidentified that all private information of patients was not included. The Institutional Review Board (IRB) of the Second Hospital of Shandong University approved the protocol of our study and stated, “since the study used historical medical records and did not involve privacy information of patients, we agreed to waive the requirement of informed consent for this study”. All procedures complied with the Declaration of Helsinki for research involving human subjects.

### Study population

From December 2015 to May 2019, 810 consecutive patients who undergone RRA at Nuclear Medicine Department, the Second Hospital of Shandong University were initially enrolled. All patients included underwent total/near-total thyroidectomy before presentation for radioiodine ablation. In addition, a prophylactic bilateral central (compartment VI) node dissection was performed for all patients with papillary carcinoma. 41 patients accepted preoperative enhanced CT investigation and the timeframe between CT investigation and RRA was from 97 to 255 days. 6–8 months after the first RRA, an initial follow-up examination was performed. Patients were excluded due to at least one of the following reasons: (1) inadequate follow-up information, (2) absence of pre-ablative TSH level collected right before ^131^I therapy; (3) distant metastases found before initial ^131^I therapy; (4) interfering antithyroglobulin antibody (TgAb) (> 46 IU/mL)^8^; (5) cases with a history of radioiodine ablation therapy; (6) large thyroid remnant (the maximum diameter of remnant tissue was more than 2 cm evaluated by ultrasound right before RRA); (6) pathological type was medullary carcinoma. Finally, total 689 patients were included in the study.

### Treatment

Between thyroidectomy surgery and RRA, L-Thyroxine 4 (LT4) treatment was stopped for 1 month. Patients were asked to follow a low-iodine diet for a month prior to RRA. All patients included had ablation with 3.70 GBq (100 mCi) ^131^I administered orally. A whole-body post-treatment scan was obtained 72 h after the administration of iodine. Patients were subsequently discharged on a TSH-suppressive dose of LT4, 100–200 μg orally per day.

### Follow-up examination

The initial follow-up was performed 6–8 months after first RRA. At the visit, all patients underwent ultrasound examination of neck, diagnostic whole-body scan (DxWBS), laboratory examination measurement including TSH, free triiodothyronine, free thyroxine, triiodothyronine, thyroxine, TgAb and TSH-stimulated or TSH-suppressed thyroglobulin (Tg).

### Laboratory examination

TSH was determined by chemiluminescence immunoassay (provided by Beckman Coulter Inc., Brea, California) with a functional sensitivity of 0.015 mIU/L. Tg and TgAb levels were determined using electro-chemiluminescence immunoassay (provided by Beckman Coulter Inc., Brea, California) with a measuring range of 0.100–500 ng/mL and 0.900–2500 IU/mL, respectively.

### Tumor-node-metastasis (TNM) staging

Based on the original pathology report, tumor-node-metastasis (TNM) staging of patients was evaluated to match the 8th edition of the AJCC/TNM staging system of thyroid cancer^9^.

### Definitions of Response to ^131^I therapy

Based on image findings and stimulated/suppressed Tg levels of the initial follow-up examination, patients were classified into four response-to-therapy categories recommended by the American Thyroid Association as shown in Table 1.

**Table 1:** Clinical implications of response to therapy reclassification in patients with differentiated thyroid cancer treated with total thyroidectomy and radioiodine remnant ablation.

Category	Definitions
Excellent response (ER)	Negative imaging and either Suppressed Tg < 0.2 ng/mL or Stimulated Tg < 1 ng/mL
Biochemical incomplete response (BIR)	Negative imaging and Suppressed Tg ≥ 1 ng/mL or Stimulated Tg ≥ 10 ng/mL or rising Anti-Tg antibody levels
Structural incomplete response (SIR)	Structural of functional evidence of disease with any Tg level with or without Anti-Tg antibodies
Indeterminate response (IDR)	Nonspecific findings on imaging studies; faint uptake in thyroid bed on RRA scanning; Non-stimulated Tg detectable, but < 1 ng/mL; Stimulated Tg detectable, but < 10 ng/mL or Anti-Tg antibodies stable or declining in the absence of structural or functional disease

### Statistical analysis

The software package R (version 3.5.3) was used for statistical analysis. For numeric variables, means and standard deviations (*SD*), or medians with interquartile ranges (*P*_*25*_, *P*_*75*_) were calculated. Independent *t*-test was used to compare the difference in numeric variables which obeyed normal distribution and Wilcoxon rank sum test was applied for numeric variables which did not obey normal distribution. For categorical variables, absolute numbers with percentages were recorded. Pearson’s Chi-squared tests or Fisher’s exact test, where appropriate, were used to compare the difference in categorical variables. For ranked data, Spearman’s correlation coefficient was used to test correlation between two variables.

Multivariable binary logistic regression model was used to explore the optimum pre-ablative TSH level range and independent factors associated with response to RRA. In the analysis, the four response-to-therapy categories were classified into a dichotomous variable including complete response (including excellent response (ER) and indeterminate response (IDR); assigned a value of 0) and incomplete response (including biochemical incomplete response (BIR) and structural incomplete response (SIR); assigned a value of 1). Univariate analysis was first used and then variables that were significant at 0.10 (*α*) level was included in the multivariate logistic model. A bidirectional stepwise elimination approach was used to simplify the model on the basis of the Alaike Information Criterion (AIC). The Variance Inflation Factor (VIF) was used to measure of multicollinearity. VIF > 10 was a criterion for judging the multicollinearity of factors.

All tests were two-sided, and a *P* value less than 0.05 was considered statistically significant. Confidence Intervals (CI) for proportions are reported as two-sided exact binomial 95% CI.

## Results

### Basic characteristics

689 patients were included in this study (499 females and 190 males; mean age, 43.22 ± 11.85). ER was observed in 364 (52.83%) patients, IDR was observed in 102 (14.80%) patients and both of them were classified into the group of complete response (n = 466, 67.63%); 55 (7.98%) patients had BIR, 168 (24.38%) patients had SIR, and both of them were classified into the group of incomplete response (n = 223, 32.37%). Six factors including gender, lymph node dissection, Tumor Stage, Node Stage, pre-ablative Tg and ATA risk classification were statistically different between complete response group (ER + IDR) and incomplete response group (BIR + SIR) with the criteria of *P* < 0.10. Details were shown in Table 2.

**Table 2:** Patients’ characteristics stratified by response-to-therapy.

Variables	Total (n = 689)	Complete response (n = 466)	Incomplete response (n = 223)	*P* value
**Gender**				** < 0.001**
Female	499(72.42)	357(76.61)	142(63.68)	
Male	190(27.58)	109(23.39)	81(36.32)	
Age at diagnosis	43.22 ± 11.85	43.20 + 11.07	43.27 + 13.37	0.946
**Thyroid operation**				0.776
Total thyroidectomy	606(87.95)	411(88.20)	195(87.44)	
Near-total thyroidectomy	83(12.05)	55(11.80)	28(12.56)	
**Lymph node dissection**				**0.004**
Lateral and central node dissection	276(40.06)	204(43.78)	72(32.29)	
Central node dissection	413(59.94)	262(56.22)	151(67.71)	
**Histology**				0.146
Papillary carcinoma	681(98.83)	463(99.36)	218(97.76)	
Follicular carcinoma	8(1.17)	3(0.64)	5(2.24)	
**Tumor stage**				**0.001**
T0	3(0.43)	3(0.64)	0(0.00)	
T1a	310(44.99)	225(48.28)	85(38.12)	
T1b	198(28.74)	139(29.83)	59(26.46)	
T2	86(12.48)	52(11.16)	34(15.25)	
T3a	8(1.16)	4(0.86)	4(1.79)	
T3b	15(2.18)	4(0.86)	11(4.93)	
T4a	3(0.43)	2(0.43)	1(0.45)	
Tx	66(9.59)	37(7.94)	29(13.00)	
**Node stage**				**0.002**
N0	95(13.79)	65(13.95)	30(13.45)	
N1a	355(51.52)	259(55.58)	96(43.05)	
N1b	239(34.69)	142(30.47)	97(43.50)	
**TNM stage**				0.304
I	583(84.62)	400(85.84)	183(82.06)	
II	105(15.24)	65(13.95)	40(17.94)	
III	1(0.14)	1(0.21)	0(0.00)	
**Pre-ablative Tg**	2.46(0.60,8.96)	1.63(0.40,5.37)	7.95(1.43,34.62)	** < 0.001**
ATA risk classification*				**0.010**
Low	41(5.95)	34(7.30)	7(3.14)	
Intermediate	569(81.28)	382(81.97)	178(79.82)	
High	88(12.77)	50(10.73)	38(17.04)	

*ATA risk classification was according to ATA guideline (2015)^5^.

Bold type indicated *P* < 0.05.

**Figure 1:**
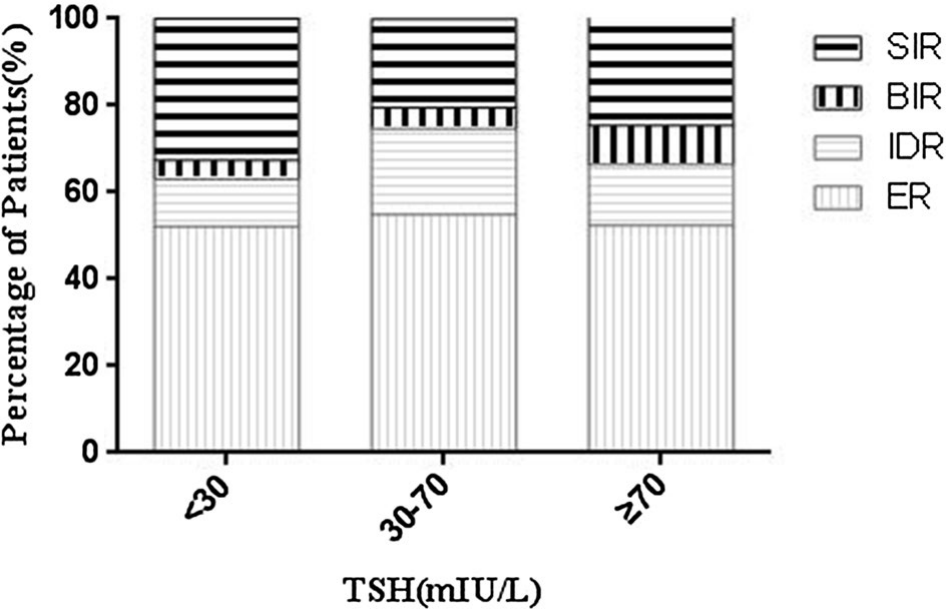
Pre-ablative TSH group and percentage of patients with response (ER, IDR, BIR and SIR) after RRA. The complete response (ER + IDR) was 63.04%, 74.59% and 66.41% separately each in three pre-ablative TSH groups of < 30, 30–70 and ≥ 70 mIU/L.

As showed in Table 3 and Fig. 1, the pre-ablative TSH level was stratified into three group (< 30, 30–70 and ≥ 70 mIU/L). Among them, 322 (46.73%) patients had the TSH level ≥ 100 mIU/L. The highest rate of complete response (ER + IDR), 74.59% was observed in the group of TSH between 30 and 70 mIU/L, whereas along with TSH increasing, the proportion of complete response was declined, 66.41% was observed in the group of TSH ≥ 70 mIU/L. Specific data of SIR was also showed in Table 3. There were 135 patients with one remnant including thyroid remnant or central lymph nodes or lateral lymph nodes in the group of SIR. The distribution of the three residual sites was roughly equal and no statistically significant difference was found between sites of residual and pre-ablative TSH levels (*P* = 0.284).

**Table 3:** Patients’ response types to RRA stratified by pre-ablative TSH levels.

Response types	Total (n = 689)	TSH (mIU/L)		
		< 30 (n = 46)	30–70 (n = 122)	≥ 70 (n = 521)
**Complete response**	466(67.63)	29(63.04)	91(74.59)	346(66.41)
ER	364(52.83)	24(52.17)	67(54.92)	273(52.40)
IDR	102(14.80)	5(10.87)	24(19.67)	73(14.01)
**Incomplete response**	223(32.37)	17(36.96)	31(25.41)	175(33.59)
BIR	55(7.98)	2(4.35)	6(4.92)	47(9.02)
SIR	168(24.39)	15(32.61)	25(20.49)	128(24.57)
Thyroid remnant	50(7.26)	8(17.39)	7(5.74)	35(6.72)
Central lymph nodes	40(5.81)	2(4.35)	7(5.74)	31(5.95)
Lateral lymph nodes	45(6.53)	2(4.35)	8(6.56)	35(6.72)
Thyroid and central lymph nodes	10(1.45)	2(4.35)	0(0.00)	10(1.92)
Thyroid and lateral lymph nodes	7(1.02)	1(2.17)	3(2.45)	6(1.15)
**Central and lateral lymph nodes**	4(0.58)	0(0.00)	0(0.00)	7(1.34)
Distant metastasis	12(1.74)	0(0.00)	0(0.00)	4(0.77)

*ER* excellent response; *IDR* indeterminate response; *BIR* biochemical incomplete response; *SIR* structural incomplete response.

According to the stratified TSH group, the study explored the relationship between different TSH levels and six factors of patients’ characteristics. All of six factors including gender, lymph node dissection, Tumor Stage, Node Stage, pre-ablative Tg and ATA risk classification were distributed differently in different groups of TSH level with the criteria of *P* < 0.10. Details were shown in Table 4.

**Table 4:** Patients’ characteristics stratified by pre-ablative TSH levels.

Variables	TSH (mIU/L)			*P* value
	< 30 (n = 46)	30–70 (n = 122)	≥ 70 (n = 521)	
**Gender**				**0.010**
Female	39(84.78)	77(63.12)	383(73.51)	
Male	7(15.22)	45(36.88)	138(26.49)	
**Lymph node dissection**				**0.083**
Lateral and central node dissection	24(52.17)	41(33.61)	211(40.50)	
Central node dissection	22(47.83)	81(66.39)	310(59.50)	
**Tumor stage**				**0.078**
T0	1(2.17)	0(0.00)	2(0.38)	
T1a	19(41.31)	63(51.64)	228(43.76)	
T1b	11(23.91)	24(19.67)	163(31.29)	
T2	5(10.87)	14(11.48)	67(12.86)	
T3a	1(2.17)	1(0.82)	6(1.15)	
T3b	1(2.17)	2(1.64)	12(2.31)	
T4a	0(0.00)	2(1.64)	1(0.19)	
Tx	8(17.40)	16(13.11)	42(8.06)	
**Node stage**				** < 0.001**
N0	18(39.13)	13(10.66)	64(12.28)	
N1a	14(30.44)	64(52.46)	277(53.17)	
N1b	14(30.43)	45(36.88)	180(34.55)	
Pre-ablative Tg	7.66(0.78,16.80)	3.01(1.02,8.50)	2.05(0.45,8.51)	**0.006**
ATA risk classification*				** < 0.001**
Low	3(6.52)	8(6.56)	30(5.76)	
Intermediate	18(39.13)	99(81.15)	443(85.03)	
High	25(54.35)	15(12.30)	48(6.97)	

*ATA risk classification was according to ATA guideline (2015)^5^.

Bold type indicated *P* < 0.05.

### Multivariate logistic regression analysis

With multivariate analysis, the study found that pre-ablative TSH levels, gender and lymph node dissection were independent predictors of response to RRA. Compared with TSH < 30 mIU/L, TSH between 30 and 70 mIU/L get more successful ablation, OR 0.451 (95% CI 0.215–0.958, *P* = 0.036). However, no statistical difference was found between the group of TSH < 30 mIU/L and TSH ≥ 70 mIU/L. Ablation was more effective in females in comparison to males, *OR* = 0.526 (95%*CI*: 0.369–0.751, *P* < 0.001). The study found multicollinearity using VIF among three factors including lymph node dissection, Node Stage and pre-ablative Tg. According to the index of AIC, we selected “lymph node dissection” into the multivariate binary logistic regression. Compared to lateral and central node dissection, only central node dissection was a risk factor for less effective RRA, which was associated with incomplete response, *OR* = 1.612 (95%*CI*: 1.149–2.276, *P* = 0.006). Moreover, it was found that there was a correlation between ATA risk classification and ascending pre-ablative TSH group tested by Spearman’s correlation coefficient (*P* < 0.001), so ATA risk classification was not included into the final multivariate model. Details were shown in Table 5.

**Table 5:** Multivariate binary logistic regression of associated factors for incomplete response (BIR + SIR) after RRA.

Variables	*β*	*OR* (95%*CI*)	*P* value
**TSH (mIU/L)**			
< 30	Ref	Ref	
30–70	− 0.795	0.451(0.215, 0.958)	**0.036**
≥ 70	− 0.285	0.752(0.401, 1.448)	0.381
**Gender**			
Male	Ref	Ref	
Female	− 0.642	0.526(0.369, 0.751)	** < 0.001**
**Lymph node dissection**			
Lateral and central node dissection	Ref	Ref	
Central node dissection	0.478	1.612(1.149, 2.276)	**0.006**

Bold type indicated *P* < 0.05.

## Discussion

After 3 to 4-week period of LT4 withdrawal, a generally accepted strategy, the TSH level of most patients not only exceeds the clinical guideline requirement of 30 mIU/L, but also is much higher than this. In our study, 75.62% of patients had the TSH level of ≥ 70 mIU/L, and the TSH exceeded 100 mIU/L in 46.73% of patients. Although all participants in the study accepted the therapy of TSH suppression and monitored the level of serum TSH every month before thyroxine withdrawal, many patients still had high level of TSH. Some other studies also showed patients had high levels of TSH. In Zhao’s study, TSH level of 64.71% (132/204) of patients applied with thyroxine withdrawal was more than 90 mIU/L^6^. There are two possible reasons: (1) the sharp and sudden decrease of thyroid hormone level in a short period of time would stimulate the rapid increase of TSH level; (2) it may be too long for patients to undergo about a month of thyroxine withdrawal. The study found that a higher level of TSH stimulation did not provide incremental benefits for DTC patients to achieve better response to initial RRA. Meanwhile, debilitating hypothyroidism caused by LT4 withdrawal would result in an at least transient loss of quality of life^10^. Further, the important function of TSH is to regulate thyroid cell proliferation, which may bring concerns that extra TSH stimulation by LT4 withdrawal might cause growth of tumor cells^11^. Thus, it is crucial to find an appropriate pre-ablative TSH level to achieve better response to RRA at the cost of relative shorter period of hypothyroidism.

In our study, the highest complete response rate of 74.59% was found in patients with a pre-ablative TSH level of 30–70 mIU/L. Beyond this level, they did not show additional benefits in terms of a better outcome. Even more, when compared with TSH < 30 mIU/L, there was no statistically significant difference on rate of response to therapy in the group of TSH ≥ 70 mIU/L. Vrachimis et al. got similar results as ours that patients with a TSH below 30 mIU/L had ablation success rates similar to those with a TSH exceeding 80 mIU/L^7^. In their study, there were no significant differences in the ablation success rates between patients with TSH < 30 mIU/L and TSH ≥ 30 mIU/L. Although the sample of their study was larger (1873 patients included), due to insufficient data quality, some essential factors like pre-ablative Tg and thyroid tissue remnant before RRA could not be evaluated. This might reduce the authenticity of their results. Lawal et al^12^. who also evaluated the relationship between pre-ablative TSH and outcome of RRA in 109 patients, found in the group with TSH level of 60–89 mIU/L the best rate of ablation, patients got the best rate of ablation. However, as TSH levels continued to rise, the ER rate declined. In Lawal’s study, the proportion of follicular thyroid carcinoma was very high (41.3%), which was different from ours (1.16% of patients were follicular carcinoma). It is recognized that patients with follicular thyroid carcinoma may have different response to RRA compared with PTC patients. From Gengpeng Li’s study, the tumor type of follicular thyroid carcinoma showed highly in dependent associations with the occurrence of radioiodine refractory^13^. So, this may be the reason why the result of Lawal’s was slightly different from ours. Another research studied by Zhao et al. found a progressively increasing ER rate with higher serum TSH levels up to 120 mIU/L in 204 non-high-risk patients with DTC^6^. Although the inflection point of TSH level for response to RRA was higher than ours, it also suggested that no additional benefits exists beyond certain TSH level.

In our study, a stronger TSH stimulation (≥ 70mIU/L) did not achieve a better response to initial RRA. A possible explanation might be the saturation of TSH receptor. From Torres, M.S.’s study, single doses of recombinant human TSH greater than 0.1–0.3 mg (the peak serum TSH concentrations was 82 ± 18 mIU/L) did not seem to further enhance thyroid hormone, which was in accordance with the concept that TSH receptors become saturated at serum TSH concentrations between 51 and 82 mIU/L^14^.

Gender and the presence of lateral lymph node dissection were also significantly associated with response to initial RRA in the study. Male gender was associated with an increase in the risk of recurrence of thyroid cancer, which was demonstrated by former studies. Jen-Der Lin et al. reported that with a mean follow-up period of 7.7 years, compared with female patients, male patients had a greater number of persistent and relapse cases^15^. In Fallahi’s study, male was an independent risk factor of successful ablation, the OR was 2.15 (95% CI 1.07–4.32)^16^. Besides, in the study patients who did not have lateral neck dissection had worse response to therapy. Maybe it was due to the existence of micro-metastasis in lateral lymph node area, which led to more remnant tumor cells postoperatively.

The study adjusted operation modes of cervical lymph node instead of Node Stage and pre-ablative Tg in the multivariate model according to the multicollinearity test. The classification of Node Stage was based on pathological result from tissue specimens of lymph nodes in the lateral cervical region, while specimens selected to examine was determined by the operation site of lymph nodes, so there was a high correlation between Node Stage and the operation mode of cervical lymph nodes. Pre-ablative Tg is an indicator of the size of remnant thyroid tissue postoperatively. There is a great correlation between the remnant thyroid tissue and the choice of surgical methods, so pre-ablative Tg also had relationship with surgical methods of cervical lymph nodes. Moreover, since the index of Tg is an important content to classify response-to-therapy of RRA, the high correlation of Tg before and after RRA may mask the effect of other factors on response-to-therapy (In other words, to some extent, the independent variable is a part of the dependent variable.). So, we did not include pre-ablative Tg in the final multivariate model.

Actually, the TSH level of most patients after four weeks of LT4 withdrawal is very high, which could not improve the therapeutic effect of initial RRA. Consequentially, many patients have possibly unnecessarily been submitted to a period of potentially debilitating hypothyroidism^10^. Novel TSH stimulation strategies for RRA are possibly needed. In patients with a rapid postoperative rise in TSH levels it could be possible to limit the period of LT4 withdrawal to 1–2 weeks^17^. Thus, the duration of hypothyroidism is limited, and quality of life may not be as badly affected. Or instead of complete LT4 withdrawal, a 4-week period of reduced LT4 substitution may reduce the debilitating side effects of deep hypothyroidism. However, further preclinical and clinical studies are required before such a strategy can be adopted in routine clinical care. Moreover, the use of recombinant human thyrotropin (rhTSH) has been adopted in many countries as an alternative to thyroxine withdrawal avoiding hypothyroidism^18^. The evaluation of therapeutic effect and cost–benefit analysis on rhTSH in China will be needed after marketing.

## Limitation

First limitation of our study is the retrospective evaluation of data. Second limitation concerns the period of follow-up considered. Studies with a longer follow-up are probably needed to assess the risk of recurrence properly.

## Conclusion

On the basis of our findings, we recommend controlling the pre-ablative TSH of 30–70 mIU/L for DTC patients, which may be of value in determining the appropriate timing of initial RRA. Further, pre-ablative TSH monitoring is necessary to determine the individual time of initial RRA administration.
